# Trans-myocardial Extraction of Endothelin-1 Correlates with Increased Microcirculatory Resistance following Percutaneous Coronary Intervention

**DOI:** 10.1155/2022/9154048

**Published:** 2022-09-19

**Authors:** George R. Abraham, Duuamene Nyimanu, Rhoda E. Kuc, Janet J. Maguire, Anthony P. Davenport, Stephen P. Hoole

**Affiliations:** ^1^Royal Papworth Hospital NHS Foundation Trust, Cambridge, UK; ^2^Division of Experimental Medicine and Immunotherapeutics, University of Cambridge, Cambridge, UK

## Abstract

**Objective:**

Coronary microvascular dysfunction (CMD) can complicate successful percutaneous coronary intervention (PCI). The potent endogenous vasoconstrictor peptide Endothelin-1 (ET-1) may be an important mediator. To investigate the mechanism, we sought to define the peri-procedural trans-myocardial gradient (TMG-coronary sinus minus aortic root levels) of ET-1 and its precursor peptide – Big ET-1. We then assessed correlation with pressure-wire indices of CMD: coronary flow reserve (CFR) and index of microvascular resistance (IMR).

**Methods:**

Paired blood samples from the guide catheter and coronary sinus were collected before and after pressure-wire-guided PCI from patients with stable angina. Plasma was analysed using a specific enzyme-linked immunosorbent assay for quantification of ET-1 peptides and correlated with pressure-wire data. Non normally distributed continuous variables are presented as median [IQR].

**Results:**

ET-1 and Big ET-1 increased post-PCI in the aorta (ET-1: 0.98 [0.76–1.26] pg/ml to 1.20 [1.03–1.67] pg/ml, *P* < 0.001 and Big ET-1: 2.74 [1.78–2.50] pg/ml to 3.36 [2.33–3.97] pg/ml, *P* < 0.001) and coronary sinus (ET-1: 1.00 [0.81–1.28] pg/ml to 1.09 [0.91–1.30] pg/ml, *P* = 0.03 and Big ET-1: 2.89 [1.95–3.83] pg/ml to 3.56 [2.66–4.83] pg/ml, *P* = 0.01). TMG of ET-1 shifted negatively compared with baseline following PCI reflecting significantly increased extraction (0.03 [−0.12–0.17] pg/ml pre-PCI versus −0.16 [−0.36–0.07] pg/ml post-PCI, *P* = 0.01). Increased ET-1 trans-myocardial extraction correlated with higher IMR (Pearson's *r* = 0.293, *P* = 0.02) and increased hyperemic transit time (Pearson's *r* = 0.333, *P* < 0.01). In subgroup analysis, mean ET-1 trans-myocardial extraction was higher amongst patients with high IMR compared with low IMR (0.73 pg/ml, SD:0.78 versus 0.17 pg/ml, SD:0.42, *P* = 0.02). There was additionally a numerical trend towards increased ET-1 trans-myocardial extraction in subgroups of patients with low CFR and in patients with Type 4a Myocardial Infarction, albeit not reaching statistical significance.

**Conclusions:**

Circulating ET-1 increases post-PCI and upregulated ET-1 trans-myocardial extraction contributes to increased microcirculatory resistance.

## 1. Introduction

Recent landmark trials in stable coronary artery disease (CAD) reinforce the imperative to identify which patients will truly benefit from percutaneous coronary intervention (PCI) [1]. It is also recognised, that despite successful epicardial revascularisation with PCI, dysfunction of the coronary microcirculation can persist. PCI can both unmask and induce coronary microvascular dysfunction (CMD) leading to adverse outcomes such as post-PCI angina and type 4a myocardial infarction (MI) [2].

Coronary guidewire-based diagnostic tests assessing the microcirculation include Coronary Flow Reserve (CFR) and Index of Microcirculatory Resistance (IMR). Impaired CFR and IMR have been shown to predict adverse outcomes in acute coronary syndromes [3], stable obstructive CAD [2], and ischemia with nonobstructive coronary arteries (INOCA) [4].

Endothelin-1 (ET-1) is an endothelial peptide crucial for physiological regulation of vasomotor tone and implicated in the pathogenesis of various coronary artery disorders. Increased plasma levels of ET-1 are associated with increased coronary [5] and systemic vasoconstriction [6] in patients with INOCA and CMD, as well as endothelial dysfunction in patients with vasospastic angina [7]. ET-1 is synthesised from an inactive precursor, Big ET-1, and released from vascular endothelial cells abluminally via a continuous constitutive pathway. This is supplemented by a complementary pathway of ET-1 release from Weibel–Palade bodies, the unique storage granules of endothelial cells, in response to various extracellular signals. A proportion of ET-1 and Big ET-1 that has escaped conversion to the mature peptide is additionally detectable in plasma. Circulating Big ET-1 does not bind to ET-1 receptors and is minimally extracted, offering a surrogate marker for ET-1 release [8].

Two ET-1 receptors are known: ET_A_ and ET_B_. Within blood vessels, ET_A_ receptors are expressed ubiquitously in smooth muscle cells in the vascular medial layer, whereas ET_B_ receptor expression is highest in endothelial cells. ET-1 binding to ET_A_ leads to a powerful and long-lasting vasopressor effect, while ET_B_ binding largely results in antagonistic effects, including clearance of circulating ET-1 and vasodilatation. In human coronary arteries, the ET_A_ subtype predominates over ET_B_ [8]. Endothelin receptor antagonists are available and licensed for use in pulmonary arterial hypertension. The ET_A_ selective antagonist, Zibotentan, is also currently being investigated as a treatment for CMD in INOCA patients as part of the precision medicine with Zibotentan in microvascular angina (PRIZE) trial [9].

The trans-myocardial gradient (TMG), defined as the concentration of ET-1 in the coronary sinus (CS), minus the concentration in the guide catheter stationed in the aortic root, can be used to directly reflect dynamic changes in ET-1 release or extraction through the coronary vascular bed. The periprocedural ET-1 profile measured using TMG has not been previously investigated in the context of post-PCI microcirculatory physiology. In this study, we aimed to define the TMG of ET-1 and any changes following PCI. We then investigated the association of this with post-PCI CMD and the occurrence of type 4a MI.

## 2. Materials and Methods

With ethical approval (REC:11/EE/0277) and written informed consent, paired blood samples from the guide catheter and coronary sinus were collected from patients with stable angina undergoing guideline-directed cardiac catheterisation before and after PCI. Plasma was then analysed using a specific enzyme-linked immunosorbent assay (ELISA) for quantification of ET-1 and Big ET-1 (R&D Systems, U.S.A).

Target vessels were interrogated before and after PCI using PressureWire *X* (Abbott Vascular, USA) derived hyperemic indices: Fractional Flow Reserve (FFR), CFR, and IMR. CFR and IMR were calculated using a thermodilution technique: after advancement of a temperature and pressure sensing guidewire into the distal third of the culprit artery, 3 mL room temperature saline boluses are injected in triplicate via the guide catheter to generate a saline transit time (Tmn) from which flow can be derived: flow = 1/Tmn. CFR is given by the resting Tmn divided by hyperemic Tmn. Corrected IMR (accounting for the contribution of collateral flow) was calculated using the formula: IMRc=Pa ×Tmn  × [(*Pd* − *Pw*)/(*Pa* − *Pw*)] (IMRc = corrected IMR, Pa = proximal pressure, Tmn = hyperemic transit time, Pd = distal pressure, and Pw = coronary wedge pressure during coronary balloon occlusion) [3]. Venous blood was sampled from a peripheral vein 12 hours after PCI to measure cardiac troponin-I (Bayer, Leverkusen, Germany).

Data were analysed using SPSS v.27 for Windows (IBM Corp., USA). Normality of continuous variable distributions was tested by one-sample Kolmogorov–Smirnov test. Normally distributed continuous variables are presented as Mean (±standard deviation). Non-normally distributed continuous variables are presented as Median (interquartile range [Q1-Q3]). Pre- and post-PCI values of ET-1 and Big ET-1 in guide catheter and CS, and pre- and post-PCI TMG were compared using Related Samples Wilcoxon Signed Rank Test. Correlations between TMG and IMRc were reported using Pearson's and Kendall's tau-b correlation coefficients with two-tailed *α* < 0.05. Patients were then coded into subgroups based on incidence of Type 4a Myocardial Infarction and using the following threshold values to define post-PCI CMD: CFR value of <2 [10] and an IMRc value ≥ 30 [11]. With respect to the latter, no consensus diagnostic criteria for IMR in post-PCI CMD for stable obstructive CAD are currently available. Previous investigators have reported on the clinical significance of various IMR cutoff values between 20 and 30 in this setting [12, 13]. Additionally, there is considerable variability in IMR amongst patients without coronary artery disease [11]. In our analysis, a higher cutoff value was chosen to decrease the risk of type 1 error. Type 4a Myocardial Infarction was defined according to the Fourth Universal Definition of Myocardial Infarction [14]. Subgroup comparisons of mean post-PCI ET-1 TMG were performed using independent samples *t*-test after the assumption of homogeneity of variances was tested using Levene's test.

## 3. Results

**Figure 1:**
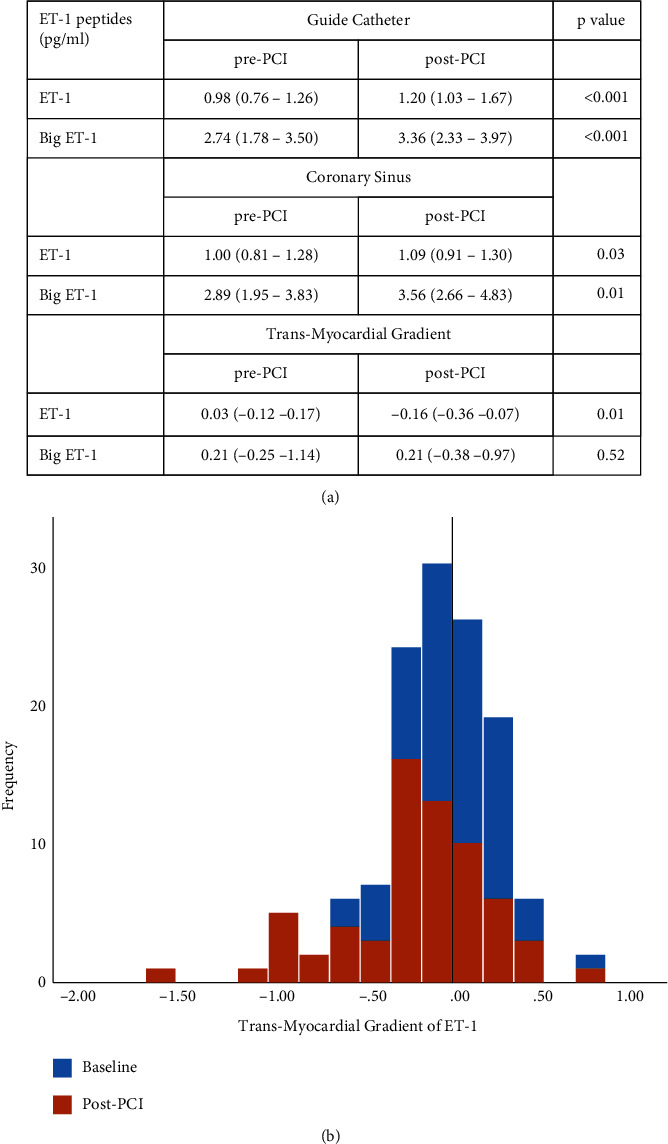
(a) Table showing median (interquartile range) of ET-1 and Big ET-1 concentrations in the guide catheter (stationed in the aortic root) and coronary sinus pre- and post-PCI. Levels of ET-1 and Big ET-1 increase significantly at both sites following PCI. (b) Histogram showing changes in the trans-myocardial gradient (coronary sinus minus guide catheter levels) of ET-1 pre-and post-PCI: trans-myocardial gradients are neutral at baseline with increased extraction (leftward shift) post-PCI.

**Figure 2:**
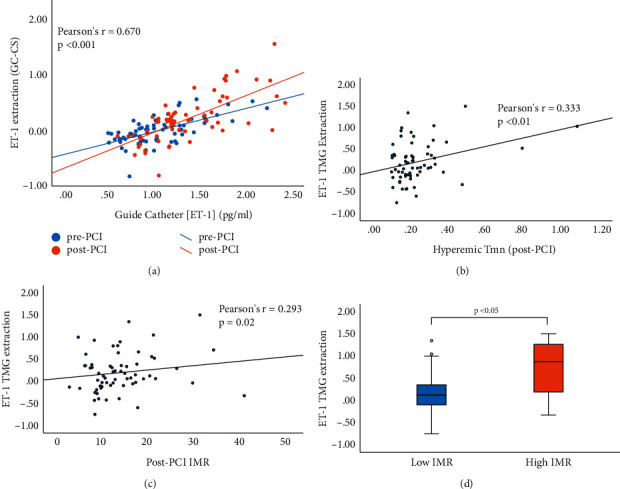
(a) ET-1 extraction (Guide catheter–coronary sinus ET-1 in pg/ml) is proportional to increased upstream ET-1 in the guide catheter stationed in the aortic root both pre-PCI and post-PCI. (b) ET-1 TMG extraction (refers to post-PCI guide catheter CS ET-1 corrected for baseline TMG by subtracting pre-PCI guide catheter-CS ET-1) correlates with decreased hyperemic flow and increased Tmn. (c) Similarly with IMRc measured post-PCI. (d) ET-1 TMG extraction is significantly higher in the subgroup of patients with post-PCI IMRc ≥30.

Paired guide catheter and CS plasma samples were obtained from 66 patients. 56 (85%) were males with a mean age of 64.5 ± 9.5. Baseline demographic characteristics are detailed in Table 1. Comparisons of pre- and post-PCI coronary physiology and further procedural details are included in the *data supplement*. Mean IMRc decreased from baseline when measured post-PCI (18.0 ± 11.3 to 15.0 ± 13.1) while mean FFR and CFR increased (FFR: 0.71 ± 0.16 to 0.93 ± 0.05 and CFR: 2.1 ± 1.4 to 2.8 ± 1.6). Plasma quantities were sufficient for assay in all cases. ET-1 and Big ET-1 concentrations increased significantly in the guide catheter and CS following PCI (Table in Figure 1(a)). TMG of ET-1 shifted negatively following PCI reflecting significantly increased extraction: 0.03 [−0.12–0.17] pg/ml pre-PCI versus −0.16 [−0.36–0.07] pg/ml post-PCI, *P* = 0.01. Contrastingly, Big ET-1 manifested positive TMG, reflecting net release, at baseline (0.21 [−0.25–1.14] pg/ml) and following PCI (0.21 [−0.38–0.97] pg/ml), *P* = 0.52 (Figure 1(b)). ET-1 trans-myocardial extraction expressed positively (guide catheter ET-1-coronary sinus ET-1) increased in tandem with the increased upstream ET-1 measured in the guide catheter both pre- and post-PCI (Zero order correlations controlling for pre- and post-PCI status: Pearson's *r* = 0.670, *P* < 0.001, Kendall's Tau-b = 0.497, *P* < 0.001Figure 2(a)). Post-PCI, ET-1 trans-myocardial extraction (after correction for baseline release or extraction) positively correlated with both hyperemic Tmn (hence decreasing flow: Pearson's *r* = 0.333, *P* < 0.01, Kendall's Tau-b = 0.174, *P* = 0.04Figure 2(b)) and post-PCI IMRc (increased resistance: Pearson's *r* = 0.293, *P* = 0.02, Kendall's Tau-b = 0.175, *P* = 0.04Figure 2(c)). There was no correlation between ET-1 trans-myocardial extraction and resting Tmn post-PCI (Pearson's *r* = 0.137, *P* = 0.275).

### 3.1. Subgroup Analysis

In subgroup analysis (Table 2), mean ET-1 trans-myocardial extraction was significantly higher amongst patients with high IMR compared with low IMR (0.73 ± 0.78 pg/ml versus 0.17 ± 0.42 pg/ml, *P*=0.02Figure 2(d)). When comparing the subgroup of patients with low CFR versus preserved CFR post-PCI, there was a numerical trend towards increased ET-1 trans-myocardial extraction albeit not reaching statistical significance (0.27 ± 0.51 pg/ml versus 0.16 ± 0.42 pg/ml, *P*=0.34). A similar trend for higher ET-1 trans-myocardial extraction was seen when comparing patients meeting criteria for Type 4a Myocardial Infarction compared to those without (0.36 ± 0.56 pg/ml versus 0.14 ± 0.40 pg/ml, *P*=0.13).

## 4. Discussion

We have shown that following PCI, the concentration of circulating plasma ET-1 peptides increases and there is upregulated ET-1 trans-myocardial extraction. Circulating ET-1 plasma concentration has previously been shown to correlate with increased vasoconstriction in patients with both obstructive coronary artery disease [24] and INOCA [5–7, 15] indicating that, despite the largely abluminal secretion of ET-1 toward vascular smooth muscle cells in the tunica media [8], ET-1 concentrations in the tissues (at least in the baseline steady state) are fairly reflected by that in the plasma. In our study of patients undergoing PCI, examination of the trans-myocardial gradient through the coronary vascular bed has revealed that increased upstream ET-1 leads to increased trans-myocardial ET-1 extraction (Figure 2(a)). As such, the true total increase in ET-1 concentration at the level of the tissues will likely be underestimated by plasma measurements. Our results indicate that upregulated ET-1 extraction is significantly associated with decreased hyperemic coronary flow and increased IMR (Figures 2(b) and 2(c)). We hypothesise that upregulated ET-1 extraction from plasma into tissues reflects increased ET_A_ receptor binding causing increased vasoconstriction in the coronary microcirculation post-PCI. Additionally, slower flow may allow more ET-1 plasma extraction, leading to a vicious cycle that increases the probability of CMD. This pathological mechanism could be treated with ET_A_ receptor antagonists.

Patients with abnormal CFR post-PCI and those suffering type 4a MI also demonstrated higher ET-1 trans-myocardial extraction, albeit not statistically significant. However these endpoints are less specific proxies for CMD and less likely to be strongly associated with ET-1 effects. For example, periprocedural MI can occur due to stent-induced side-branch occlusion. Compared with IMR, CFR is more easily perturbed by prevailing hemodynamics while submaximal reactive hyperemia post-PCI can transiently increase resting coronary flow and paradoxically decreasing CFR [16]. This may underpin the lack of correlation observed between resting Tmn and ET-1 trans-myocardial extraction as compared with hyperemic Tmn.

Increased post-PCI ET-1 has been reported in the coronary circulation by multiple investigators following balloon angioplasty for stable CAD [17–19]. Increased trans-myocardial ET-1 extraction (between coronary artery and coronary sinus) has also been previously reported by Petronio et al. [18]. We extend these findings by demonstrating deleterious effects on the microcirculation. Our results highlight a dynamic relationship between ET-1 concentration and plasma extraction following PCI. Therefore, it is likely that differences in the timing and technique of blood sampling as well as the nature of the coronary intervention performed (balloon angioplasty only versus stenting procedures) explains the inconsistency of overall ET-1 trans-myocardial gradients reported in earlier studies [17–19].

**Figure 3:**
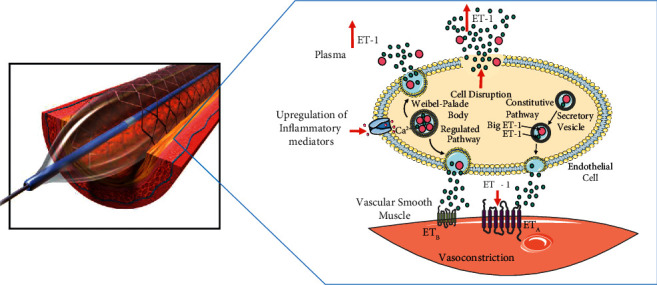
Suggested scheme for upregulation of ET-1 post-PCI. Mechanical disruption of atherosclerotic plaque and endothelial cells by balloon angioplasty leads to release of ET-1 and Big ET-1. Additionally, increased circulating inflammatory mediators post-PCI may upregulate the regulated Weibel–Palade Body secretory pathway. ET-1 binds to ETA receptors on vascular smooth muscle cells causing vasoconstriction. Figure incorporates material derived from angioplasty: balloon inflated with stent by Blausen Medical used under license: CC BY-SA 4.0 and Servier Medical Art (smart.servier.com) used under License CC BY 3.0.

Disruption of cells containing ET-1 in atherosclerotic plaque is a possible explanation for the overall increase in circulating ET-1 post-PCI. ET-1 and Big ET-1 have been previously detected in macrophages, myointimal cells, myofibroblasts, and endothelial cells, as well in the extracellular space of coronary atheromatous tissue examined following atherectomy [19]. With respect to the increased ET-1 measured in the guide catheter, upstream of the plaque, we hypothesise systemic inflammatory factors induced during PCI may upregulate the stimulated ET-1 pathway (Figure 3). This is consistent with previous studies demonstrating increased ET-1 post-PCI in peripheral vessels [20–22]. Alternatively (especially in the case of more proximal coronary interventions), aortic root ET-1 may have been influenced by blood sampling from the proximal coronary artery. Further clarification could be obtained in the future by *trans-coronary* rather than trans-myocardial ET-1 sampling [23].

### 4.1. Limitations

Our study was a small single-centre observational study with multiple correlations analysed; therefore, the data should be viewed as hypothesis-generating only. We recognise that correlative analyses on a narrow range of post-PCI IMR may lead to errors, although we would have expected this narrow range to bias towards a null outcome. Few patients in our cohort met the specified IMR criteria for CMD in subgroup analysis, and further validation of our findings is required in more patients with clinically significant CMD.

## 5. Conclusions

Following PCI in patients with stable angina, circulating ET-1 peptides increase. There is a commensurate increase in trans-myocardial ET-1 extraction, the extent of which correlates with increased index of microcirculatory resistance post-PCI. These results suggest a theoretical rationale for the adjunctive use of ET_A_ receptor antagonists as a tool to optimise microvascular function following PCI.

## Figures and Tables

**Table 1:** Baseline demographic details: data represent *n* (% of total) for categorical variables and mean ± SD for continuous variables.

Demographics	*n* = 66
Age	64.5 ± 9.5
Male gender	56 (83.6)
BMI	28.7 ± 5.3
LVEF ≤45%	3 (4.5)
Hypertension	27 (40.3)
DM	8 (11.9)
Hypercholesterolemia	21 (31.8)
Smoker	14 (21.2)
Family history of CAD	21 (31.8)
Previous MI	40 (60.6)
Previous PCI	35 (53)

**Table 2:** Subgroup analysis.

	Subgroups		*t* (d*f*)	*P* value
	High IMR (*n* = 4)	Low IMR (*n* = 61)		
ET-1 TMG extraction (pg/ml)	0.73 ± 0.78	0.17 ± 0.42	2.5 (63)	0.02
	Low CFR (*n* = 26)	High CFR (*n* = 39)		
ET-1 TMG extraction (pg/ml)	0.27 ± 0.51	0.16 ± 0.42	−1.0 (63)	0.34
	Type 4a MI (*n* = 19)	No type 4a MI (*n* = 46)		
ET-1 TMG extraction (pg/ml)	0.36 ± 0.56	0.14 ± 0.40	−1.6 (26.0)	0.13

High IMR refers to post-PCI IMRc ≥30 and low CFR refers to post-PCI CFR values < 2. ET-1 TMG extraction is presented as mean ± SD; and t(df) refers to two sample t-value with degrees of freedom.

## Data Availability

All data that support the findings of this study are available from the corresponding author upon reasonable request.

## Supplementary Materials

Supplementary Materials A data supplement is available attached as a separate file. The data supplement contains additional demographic and coronary physiology data (Table S1) and procedural characteristics (Table S2).
