# Supplementary material for: Trans-myocardial Extraction of Endothelin-1 Correlates with Increased Microcirculatory Resistance following Percutaneous Coronary Intervention
Source: J Interv Cardiol. 2022 Sep 19;2022:9154048. doi: 10.1155/2022/9154048 (PMC9553718; doi:10.1155/2022/9154048)
Supplement: Supplementary Materials — A data supplement is available attached as a separate file. The data supplement contains additional demographic and coronary physiology data (Table S1) and procedural characteristics (Table S2). [file 9154048.f1.docx]

**Data Supplement**

All data that support the findings of this study are available from the corresponding author upon request.

**Table S1:** Comparison of physiological data (mean±SD) from 67 interrogated vessels from 66 patients. A paired samples t-test was used to compare baseline and post-PCI FFR, CFR and IMRc.

| **Physiological data (n=67)** | Baseline | Post-PCI | p value |
| --- | --- | --- | --- |
| FFR | 0.71±0.16 | 0.93±0.05 | <0.001 |
| CFR | 2.1±1.4 | 2.8±1.6 | <0.001 |
| IMRc | 18.0±11.3 | 15.0±13.1 | 0.045 |
| CFIp | 0.12±0.13 |  |  |
| RAP (mmHg) | 6.2±2.9 |  |  |

**Table S2:** Details of the PCI procedures including stent type, length and diameter of stent implanted and target vessel for intervention.

| **Procedural characteristics** | n=67 |
| --- | --- |
| Radial Access | 60 (90.9) |
| DES | 38 (56.7) |
| BVS | 23 (34.3) |
| BMS | 7 (10.4) |
| Stent length | 34.9±16.5 |
| Stent diameter | 3.4±0.5 |
| **Target Vessel** |  |
| LAD | 40 (59.7) |
| LCx | 9 (13.4) |
| RCA | 15 (22.4) |
| Intermediate/OM | 3 (4.5) |

**Table S2 legend:** Continuous variables are represented by mean±SD and categorical variables by n (% of total). DES refers to Drug eluting stents, BVS: Bioresorbable vascular scaffolds, BMS: bare metal stents. LAD refers to left anterior descending artery, LCx: left circumflex artery, RCA: right coronary artery, OM: oblique marginal artery.
